# A new advance in alternative splicing databases: from catalogue to detailed analysis of regulation of expression and function of human alternative splicing variants

**DOI:** 10.1186/1471-2105-8-180

**Published:** 2007-06-04

**Authors:** Pierre de la Grange, Martin Dutertre, Margot Correa, Didier Auboeuf

**Affiliations:** 1 INSERM U685/AVENIR, Centre G. Hayem, Hôpital Saint-Louis, 1 avenue Claude Vellefaux, 75010 Paris, France

## Abstract

**Background:**

Most human genes produce several transcripts with different exon contents by using alternative promoters, alternative polyadenylation sites and alternative splice sites. Much effort has been devoted to describing known gene transcripts through the development of numerous databases. Nevertheless, owing to the diversity of the transcriptome, there is a need for interactive databases that provide information about the potential function of each splicing variant, as well as its expression pattern.

**Description:**

After setting up a database in which human and mouse splicing variants were compiled, we developed tools (1) to predict the production of protein isoforms from these transcripts, taking account of the presence of open reading frames and mechanisms that could potentially eliminate transcripts and/or inhibit their translation, i.e. nonsense-mediated mRNA decay and microRNAs; (2) to support studies of the regulation of transcript expression at multiple levels, including transcription and splicing, particularly in terms of tissue specificity; and (3) to assist in experimental analysis of the expression of splicing variants. Importantly, analyses of all features from transcript metabolism to functional protein domains were integrated in a highly interactive, user-friendly web interface that allows the functional and regulatory features of gene transcripts to be assessed rapidly and accurately.

**Conclusion:**

In addition to identifying the transcripts produced by human and mouse genes, fast DB  provides tools for analyzing the putative functions of these transcripts and the regulation of their expression. Therefore, fast DB has achieved an advance in alternative splicing databases by providing resources for the functional interpretation of splicing variants for the human and mouse genomes. Because gene expression studies are increasingly employed in clinical analyses, our web interface has been designed to be as user-friendly as possible and to be readily searchable and intelligible at a glance by the whole biomedical community.

## Background

Human genes are transcribed as messenger RNA precursor molecules (pre-mRNAs), which are composed of short exons separated by much longer introns. The introns are removed during the splicing process, which gives rise to mature mRNAs containing only exons. Genome-wide analyses indicates that most (up to 70%) human genes generate different transcripts with different exon contents by using alternative promoters, alternative polyadenylation sites and alternative splice sites [1-3]. About 40% of human genes produce at least five different splicing variants (SVs) and up to 10% of them produce more than 10 alternate transcripts each [4,5]. With such diversity and complexity, it becomes difficult to apprehend the role and impact of the transcriptome in terms of cellular function. A first step was attained by several large-scale studies showing that 75% of alternative splicing (AS) events occur in translated regions of mRNAs and have consequences at the protein level. The resulting changes in amino acid sequence can alter the binding properties of proteins, influence their intracellular location, and modify their enzymatic activity and/or stability [2]. This leads to a gain, an alteration, or even a complete loss of function. Importantly, although a given transcript can be predicted to produce a particular protein isoform, its translation can be inhibited by several mechanisms or it can be degraded. These mechanisms include nonsense-mediated mRNA decay (NMD) [6-8] and targeting of microRNAs (miRNA) [9-11]. The NMD pathway leads to the degradation of mRNAs that contain a premature stop codon, i.e. a stop codon more than 50 nucleotides upstream of any exon/exon junction [6,7]. MiRNAs are 21- to 25-nucleotide-long RNAs that can either induce the degradation or suppress the translation of mRNAs, depending on whether they match their targets perfectly or approximately [9]. Several databases based on different algorithms able to predict miRNA targets have been constructed [12-14]. In addition to considering the production of protein isoforms from SVs, biologists are interested in their expression patterns. Global gene expression, promoter use and SV expression can be specific to a tissue or a group of tissues [15,16]. Therefore, to apprehend transcriptome diversity and complexity, it is now necessary to develop interactive databases that provide tools for predicting the functions of SVs as well as their tissue expression patterns.

Having compiled human and mouse splicing variants in a database named fast DB [4], we now provide resources for functional interpretation of SVs. Indeed, this new release of fast DB provides a predicted open reading frame (ORF) for each known transcript, and helps predict the functional consequences of AS events by an analysis of protein domains encoded by alternative exons. Moreover, fast DB now predicts whether transcripts are potential targets for an NMD pathway and/or miRNA. Finally, this new version of fast DB provides tools for studying the expression and regulation of SVs. In particular, fast DB now provides tissue distribution charts of each gene and AS event to help study the tissue specificity of AS events.

## Construction and content

### Definition of exons and AS events

#### Genomic sequence and orthologous relationships

We recovered human genomic sequences of the 22,218 "protein coding" genes from the homo_sapiens_core_31_35d EnsEMBL database [17]. Mouse genomic sequences came from the mus_musculus_core_35_34d EnsEMBL database. We used the ensemble_compara_36 database to associate each human gene with its orthologous mouse gene.

#### Transcript selection

To define exon/intron structures and alternative splicing events, we aligned transcript sequences against genomic sequence using sim4 [18]. "Full length" mRNAs and expressed sequence tags (ESTs) came from the UCSC website as available in January 2006 [19] and "partial" mRNAs were downloaded from the NCBI website [20]. Transcripts were selected using very stringent criteria (see the fast DB documentation).

#### Definition of "genomic exons" and AS events

We defined a "genomic exon" as the most frequently-occurring transcript exon at a given genomic position. Alternative events were defined by comparing transcript exons with the corresponding genomic exons (see the fast DB documentation). Statistics of AS events in fast DB are available on the fast DB website.

### Known coding sequences

To display information about known translation product(s), fast DB shows the known coding sequence(s) (CDS) for most human genes. We downloaded data from the Consensus CDS database (CCDS) as available on March 2, 2005 [21]. Using EnsEMBL transcript accession (e.g. ENST00000343008), we associated 12,201 fast DB human genes with at least one CDS from the CCDS database, which contains CDS from 13,142 human genes. On average, each fast DB human gene was associated with 1.1 CDS from the CCDS database.

### Prediction of open reading frames

To help predict the impact of AS on protein function, fast DB presents interactive graphical representations of ORFs for most human and mouse cDNAs. In some cases, two ORFs are predicted for the same SV (Figure 2, items 5 of [GenBank:BC063849]). We used the "getorf" program from the EMBOSS package [22] to find all ORFs of at least 120 nucleotides ("getorf -minsize 120 -reverse No file_in file_out"). We then selected the two longest ORFs. The first of these (blue ORFs in Figure 2, items 5) corresponds to the ORF covering the greatest number of transcript exons. If there are AS events before or within the first exon covered by the first selected ORF, fast DB displays the second (red ORF of the [GenBank:BC063849] transcript in Figure 2, item 5). Using this algorithm, fast DB provides ORF predictions for 111,893 human cDNAs (99.4% of the 112,609 human cDNAs) and 76,391 mouse cDNAs (97.7% of the 78,170 mouse cDNAs). Two predicted ORFs are displayed for 8,450 (7.5%) human cDNAs and for 4,780 (6.1%) mouse cDNAs.

### Prediction of nonsense-mediated mRNA decay

To predict whether a given transcript is a target for the NMD pathway, we calculated nucleotide length between the stop position (on the transcript sequence) and the position of the last exon-exon junction. If this length is greater than 50, the corresponding SV is predicted to be targeted by NMD. Fast DB labels each predicted ORF with an "ORF" flag, or with an "NMD" flag if NMD targeting is predicted. Among all human cDNAs, 13% (14,667 of 112,609) have at least one transcript labeled "NMD" [14,667 (31%) of 47,923 of alternate human cDNAs].

### Prediction of microRNA/transcript interaction sites

To predict whether a given exon is a potential target for a miRNA, we downloaded a file with all predicted miRNA/transcript interaction sites from the miRBase Targets database version 2.0 [13]. This file contains miRNA names, chromosome names and chromosomal positions. From these positions, we realigned the miRNA sequences with corresponding genomic sequences using the miRanda program [23]. Interaction sites with a maximum energy of -19 were stored in the fast DB MySQL database [24]. Each transcript sequence within the alignment region was aligned with the genomic sequence using Clustalw [25], and differences between genomic and transcript sequences were highlighted in red in order to predict whether they potentially affect miRNA/transcript interaction. A total of 78,903 miRNA/transcript interaction sites were predicted between 11,301 human genes (63% of the 18,008 human genes) and 413 microRNAs.

### Association of transcript with tissue

To provide a tissue-specificity analysis of each AS event, we associated each human transcript (cDNAs and ESTs) with the tissue from which it had been cloned. Where information was available, we recovered the name of the transcript library from CGAP [26], and where the library was associated with a tissue, we associated tissue with transcript using a keyword search system written in Perl [27] among a collection of 36 tissues and groups of tissues (see the fast DB documentation). If a transcript was not associated with a library, or if its library was not associated with a tissue, we used our keyword search system on the "tissue_type" field of the transcript GenBank file (where this field was available). Using this algorithm, 87% of fast DB transcripts were associated with one of the 36 tissues. Among the 1,154,554 transcripts stored in the fast DB database, 875,479 (76%) were associated with a tissue using the library information and 132,740 (11%) were associated with a tissue using their "tissue_type" information. A histogram of tissue distribution of all the fast DB cDNAs and ESTs is available in the fast DB documentation and on the fast DB website (Figure 5A, item 1).

### Tissue distribution histogram of all gene transcripts

To enable the expression pattern of a given gene to be visualized clearly, fast DB displays a histogram of the tissue distribution of all its transcripts. Once the transcripts had been associated with the tissue from which they were cloned, we used the Perl module GD::Graph to draw this histogram. Transcripts not associated with a tissue were placed in the "n/a" group. Only tissues in which transcripts are expressed were represented on the chart.

### Tissue distribution histogram of gene transcripts for a specific event

To go further, fast DB provides a histogram of the tissue distribution of transcripts for each transcriptional and splicing event.

#### Analysis of alternative first exons

All the different alternative first exons were represented on the same chart. For each alternative first exon, we identified the transcripts for which this event was defined. All 5'-partial transcripts were excluded from this study, i.e. transcripts for which no alternative first exon was defined (see the fast DB documentation).

#### Analysis of alternative terminal exons

As for the alternative first exons, all the different alternative terminal exons were represented on the same chart. For each alternative terminal exon, we identified the transcripts for which this event was defined. All 3'-partial transcripts were excluded from this study, i.e. transcripts for which no alternative terminal exon was defined (see the fast DB documentation).

#### Exon skipping

Several groups of transcripts were represented on the chart. The first group comprises transcripts for which exon skipping is defined (see the fast DB documentation). The next group comprises transcripts that include the corresponding exon(s). The last group can be divided into subgroups according to splicing events defined as adjacent to the event studied. For this purpose, we defined a pair of values corresponding to the positions of splice sites flanking the skipped exon(s) (Figure 5B, items 2). For each pair of values, a group was represented on the chart, in addition to the group of transcripts for which exon skipping was defined.

#### Intron retention

As for exon skipping, several groups of transcripts were represented on the chart. The first group consists of transcripts for which intron retention is defined (see the fast DB documentation). The other consists of transcripts that splice introns (genomic or nongenomic introns), which are included in transcripts defining the alternative event. For a single chosen intron, four values were defined (corresponding to the splice sites of the exons flanking the chosen intron). For each group of values, a transcript group was represented on the chart, in addition to the group of transcripts for which intron retention was defined.

#### Alternative 3'and 5' splice sites

Several distinct groups of transcripts were represented on the chart. Each group corresponds to a different pair of acceptor/donor splice sites (see the fast DB documentation).

## Utility

**Figure 1:**
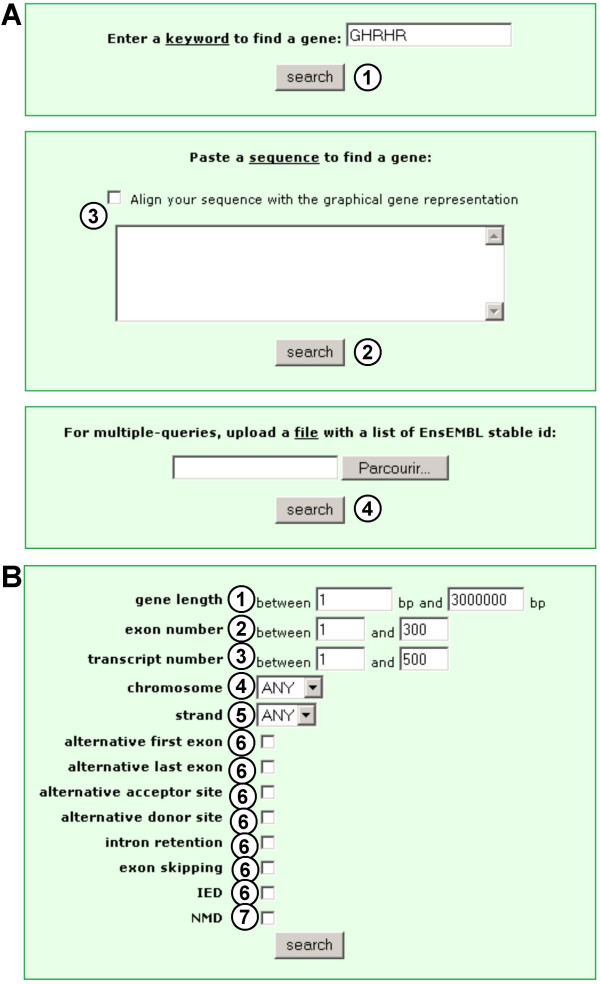
**The fast DB search pages**. (A) The fast DB basic search page. (1) Keyword search; (2) Blast search; (3) checkbox to align an input sequence with a graphical representation of the exon/intron gene structure; (4) multiple-queries search: the user can upload a file with a stable gene EnsEMBL ID at each line. (B) The fast DB advanced search page. (1) Minimal and maximal length selection in base pairs; (2) selection of minimal and maximal number of exons; (3) selection of minimal and maximal number of cDNAs; (4) chromosome selection; (5) chromosome strand selection; (6) alternative transcriptional and splicing event selection; (7) NMD pathway targeting prediction selection.

**Figure 2:**
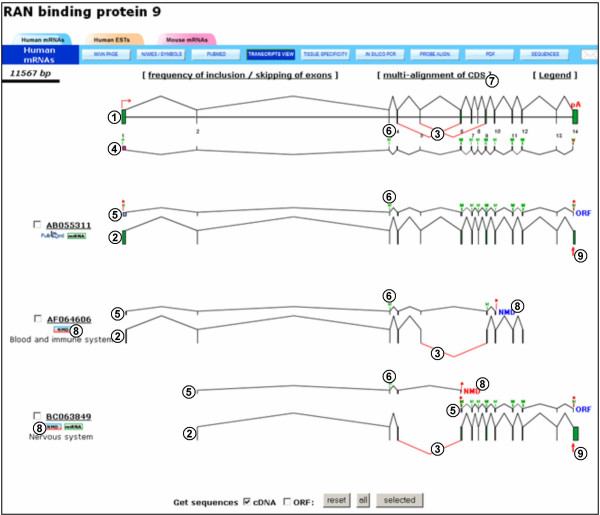
**The fast DB "transcripts view" page of theRANBP9 human gene**. (1) Graphical representation of the exon/intron gene structure. (2) Graphical representation of exon content of each transcript aligned with graphical representation of the gene. (3) AS events are highlighted in red. (4) Known CDS from the CCDS database. (5) Predicted ORFs. (6) Methionine positions are represented by a green "M". (7) Link to a multi-alignment of all translated ORF sequences. (8) Predicted NMD. (9) Exon with at least one predicted microRNA/transcript interaction site.

Fast DB can be searched using various criteria such as gene, transcript or protein IDs, keywords, and chromosomal location (Figure 1A, item 1). A blast search is available (Figure 1A, item 2), and a list of genes can also be retrieved by uploading a file gathering the stable EnsEMBL ID of these genes (Figure 1A, item 4 and see the fast DB documentation). A list of genes with similar characteristics can also be retrieved using the fast DB advanced search page (Figure 1B). The criteria available for gene selection include gene length (Figure 1B, item 1), number of exons (Figure 1B, item 2), number of transcripts (Figure 1B, item 3), chromosomal location (Figure 1B, items 4 and 5), type of AS event (Figure 1B, item 6), and NMD-targeted transcript prediction (Figure 1B, item 7). After a specific gene is selected, its main page presents its exon/intron structure and general information regarding splicing of its transcripts [4]. From there, three kinds of analyses are available, based on human mRNAs, human mRNAs and ESTs, and mouse mRNAs. After choosing one option, the user can click on the "transcripts view" button. The "transcripts view" page (Figure 2) provides a diagram of the exon/intron structure of the gene (Figure 2, item 1) and the exon content of its transcripts (Figure 2, items 2), with AS events in red (Figure 2, items 3).

Of the 18,008 human genes present in our database, 11,071 (61%) were estimated to undergo AS. This result is consistent with other studies, which estimate that 40–75% of human genes are alternatively spliced [1-3,28,29]. Using 112,609 cDNAs mapped to the human genome, we defined 5,663 cassette exons, 3,940 intron retentions, 6,483 alternative acceptor sites and 5,551 alternative donor sites. Finally, 26% and 22% of the fast DB genes present alternative first and alternative terminal exons, respectively. These results are highly consistent with those of other databases [5,30-35]. More detailed statistics can be found on the fast DB website.

### Prediction of protein isoform production from gene transcripts in fast DB

#### Prediction of alternative coding sequence functions

**Figure 3:**
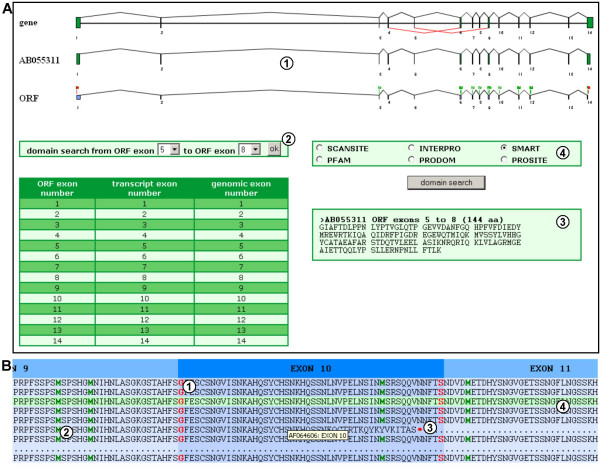
**Functional protein domain analysis andmulti-alignment of the translated sequences of predicted ORFs**. (A) Functional protein domain analysis of the [GenBank:AB055311] transcript. (1) Diagram of the [GenBank:AB055311] ORF aligned with the corresponding transcript and gene graphical representations. (2) Form for selecting ORF exons for analysis. (3) Translated sequences of the selected ORF exons. (4) Form for selecting external websites to predict functional protein domains. (B) Multi-alignment of translated sequences of predicted ORFs. (1) Amino acids encoded by nucleotides from two exons (the amino acid is within the exon that yields two of the three codon nucleotides). (2) Methionines are represented by a green bold "M". (3) Red bold asterisks correspond to stop codons. (4) CDS from CCDS is highlighted in green.

Fast DB integrates information and tools for predicting the functional consequences of AS. To do that, fast DB first displays the known CDS(s) under the gene representation (Figure 2, item 4) and provides a predicted ORF for most transcripts (Figure 2, items 5). The known CDS(s) can be clicked on to open the corresponding CCDS page on the NCBI website. In order to compare transcript ORF frames with each other and/or the frame of known CDS(s), methionine positions are indicated on the ORF charts (Figure 2, items 6). Methionines also indicate potential translation initiation sites, as translation can be initiated from downstream in-frame AUG codons [36]. AS modifies the amino acid composition and therefore changes functional and/or structural protein domains by inserting, altering or completely deleting a domain. To help the user predict the effect of AS on protein function, each exon, particularly alternatively spliced exons, can be submitted directly to external functional protein domain prediction tools. The user just clicks on the transcript ORF of interest to open the corresponding analysis page (Figure 3A). This page shows a diagram representing the selected ORF, graphically aligned under the corresponding transcript and gene locus (Figure 3A, item 1). As the graphical representation of the gene shows AS events in red, the user can easily select alternative exons to analyze (Figure 3A, item 2). Once the choice of exon is validated, the corresponding amino acid sequence is displayed (Figure 3A, item 3), and the user can select an external website to predict the functional domain encoded within the selected exon [37-42] (Figure 3A, item 4). In addition to AS, short variations in nucleotide sequence due to polymorphisms or mutations can lead to a frameshift or to a single amino acid deletion, insertion or substitution. In order to visualize these potential amino acid polymorphisms, fast DB provides a multi-alignment of all translated sequences of predicted ORFs and known CDS(s), exon by exon (Figure 3B). Amino acids encoded within an exon/exon junction are shown in red (Figure 3B, item 1), methionines in green (Figure 3B, item 2); stop codons are shown with a red asterisk (Figure 3B, item 3), amino acid polymorphisms are highlighted yellow (data not shown), and known CDSs are highlighted light green (Figure 3B, item 4).

#### Prediction of no protein production: NMD pathway and miRNA targeting

It is now recognized that many transcripts are not translated, or are poorly translated, owing to rapid degradation and/or translational repression through the NMD pathway and/or miRNA targeting. It is therefore important to predict whether transcripts are likely to be subject to such regulations. In addition, to diversify the proteome by changing the protein domain composition, AS may modulate gene expression by generating NMD-targeted variants. Therefore, in fast DB, transcripts that are predicted to be targeted by NMD (the stop codon is located more than 50 nucleotides upstream of the most downstream exon/exon junction) are indicated and labeled by an "NMD" close to their stop codon (Figure 2, items 8).

By analyzing 18,008 genes in fast DB, we found that 14,667 (31%) of 47,923 SVs were potential targets for the NMD pathway, which is consistent with a previous report [8]. However, this number is an underestimate because mRNAs targeted by the NMD pathway are certainly underrepresented in cDNA databanks owing to their low level of expression and to the high proportion of 3'-partial transcripts potentially targeted by the NMD pathway.

**Figure 4:**
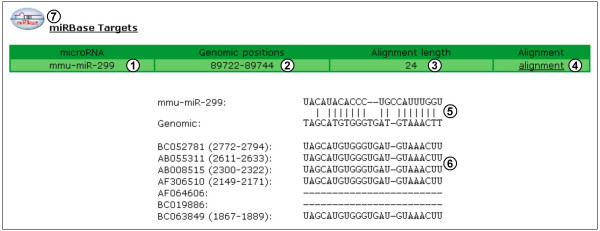
**MicroRNA interaction site prediction**. (1) Name of microRNA. (2) Positions of alignment between microRNA and genomic sequences. (3) Length of alignment between microRNA and genomic sequences. (4) Link to alignment between microRNA and genomic sequences. (5) MicroRNA sequence aligned with genomic sequence. (6) Transcript sequences aligned with the genomic sequence located at the same alignment position.

In addition to predicting whether a transcript is targeted by the NMD pathway, the fast DB "transcripts view" indicates whether a given transcript is potentially targeted by miRNAs. On the "transcripts view" page, a red arrow is displayed under a transcript exon in which at least one miRNA/transcript interaction site is predicted (Figure 2, items 9). By clicking on this arrow, a predicted miRNA/transcript interaction site (or a list of sites in some cases) is provided, with the name of the miRNA (Figure 4, item 1), the genomic position of the corresponding alignment (Figure 4, item 2), the length of this alignment (Figure 4, item 3) and the link to its display (Figure 4, item 4). MiRNA and genomic sequences are aligned with each other (Figure 4, item 5), and fast DB presents a multi-alignment of the corresponding transcript sequences (Figure 4, item 6). Where they occur, differences between genomic and transcript sequences are highlighted in red to predict whether they potentially affect miRNA/transcript interaction (data not shown). The effect of miRNA interaction on mRNA metabolism (i.e. degradation or translation repression) depends on the extent of complementarity. For more information on a given predicted miRNA/transcript interaction site, fast DB provides a link to the corresponding miRBase Targets webpage (Figure 4, item 7). Bioinformatic prediction of transcripts being targeted by miRNAs should be viewed with caution. First, miRNA target genes cannot yet be predicted with complete reliability. Second, regulation by miRNAs is strictly dependent on the expression of such miRNAs in the cell, which must be tested experimentally.

**Figure 5:**
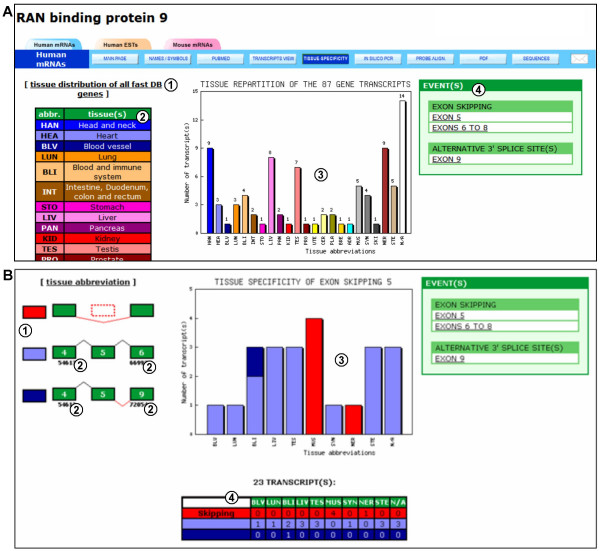
**Tissue specificity analysis in fast DB**. (A) Tissue distribution of the 87 transcripts (cDNAs + ESTs) of the human *RANBP9 *gene. (1) Link to the tissue distribution of all fast DB cDNAs and ESTs. (2) Color legend and abbreviations of tissues in which transcripts are expressed. (3) Histogram of the tissue distribution of the human *RANBP9 *gene transcripts. (4) List of alternative events available for tissue specificity analysis. (B) Tissue specificity analysis of the skipping of human *RANBP9 *gene exon 5. (1) Color legend and schematic representation of the different transcript groups constituted for the study of this event. (2) Splice site positions used to define the different groups of transcripts. (3) Histogram of transcript tissue distribution: the skipping of exon 5 (red) seems to occur specifically in muscular (and possibly nervous) tissue(s). (4) Table summarizing the tissue distribution values for the transcript.

### Advanced analysis of the regulation of transcript expression in fast DB

#### Prediction of tissue distribution and regulatory features

The expression of SVs may depend on cell type, and some SVs are only present in a specific tissue or group of tissues [43]. For each human gene, fast DB provides the tissue distribution of its transcripts (cDNAs and ESTs) (Figure 5A, item 3). Furthermore, for each alternative promoter, alternative polyadenylation site and AS event, fast DB provides the tissue distribution of the SVs defining the event (Figure 5B). For example, two distinct exon skipping events were defined for the human *RANBP9 *gene: skipping of exon 5 and skipping of exons 6 to 8. All AS events are listed on the right side of the "tissue specificity" page (Figure 5A, item 3). By clicking on a given event (e.g. skipping of exon 5), fast DB displays the tissue distribution histogram for the SVs that contain or do not contain exon 5 (in blue and red, respectively). The group of SVs containing exon 5 is divided into two subgroups according to splicing events defined adjacently to the studied event; the first subgroup corresponds to SVs that include exon 5 without defining other events (light blue), and the second corresponds to SVs that include exon 5 but do not include exons 6 to 8 (dark blue). These different subgroups of SVs are schematically represented on the left side of the page (Figure 5B, item 1). The tissue distribution chart shows that skipping of *RANBP9 *exon 5 occurs specifically in muscular (and possibly nervous) tissue(s) (Figure 5B, item 3). A table with the number of transcripts defining the events in the different tissues is presented (Figure 5B, item 4).

**Figure 6:**
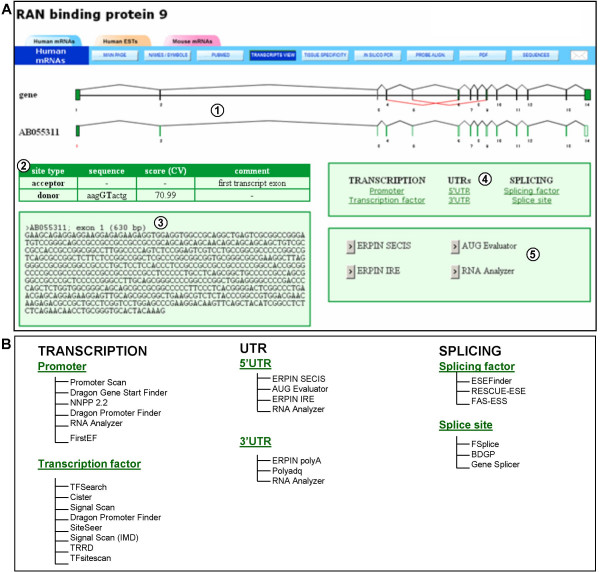
**Analysis of the first exon of the **[GenBank:AB055311] **transcript**. (A) The fast DB page for the analysis of the first exon of the [GenBank:AB055311] transcript. (1) Graphical representation of the [GenBank:AB055311] transcript aligned with the exon/intron structure of the human *RANBP9 *gene. Exon 1 was selected for analysis and labeled in red. (2) Scoring of splice site strength for the first exon of the [GenBank:AB055311] transcript. (3) Length and sequence of the first exon of [GenBank:AB055311]. (4) Analyses available for the selected transcript exon: transcription, UTR or splicing analyses. (5) Website selection for analyzing the selected exon for 5'UTR sequences. (B) List of websites available for analysis.

To provide further support for analyses of the regulation of transcript expression, each transcript exon from the "transcripts view" page (Figure 2, items 2) can be clicked on to analyze its sequence directly. This enables either promoter, transcription factor or splicing factor binding site to be predicted, or splice sites strengths to be scored, and for 5' and 3' UTR sequences to be analyzed, since RNA sequences regulating mRNA metabolism are usually located in UTRs [44]. After an exon is clicked on, a new page opens. Figure 6A presents the analysis of the first exon in the [GenBank:BC063849] transcript of the human *RANBP9 *gene. A table shows the sequences and scores of splice sites corresponding to the selected exon (Figure 6A, item 2). In this case, since the selected exon is the first in the transcript, the acceptor site is not defined. The sequence and length of the exon are also displayed under this table (Figure 6A, item 3). The user can select several kinds of analyses to proceed with this exon (Figure 5A, item 4): transcription analyses, UTR analyses or splicing analyses. Once the kind of analysis is selected (5' UTR analyses in this example), the user must choose an external website to perform it (Figure 6A, item 5). All the possible websites are listed in Figure 6B.

#### Tools to assist in experimental analysis of SV expression

**Figure 7:**
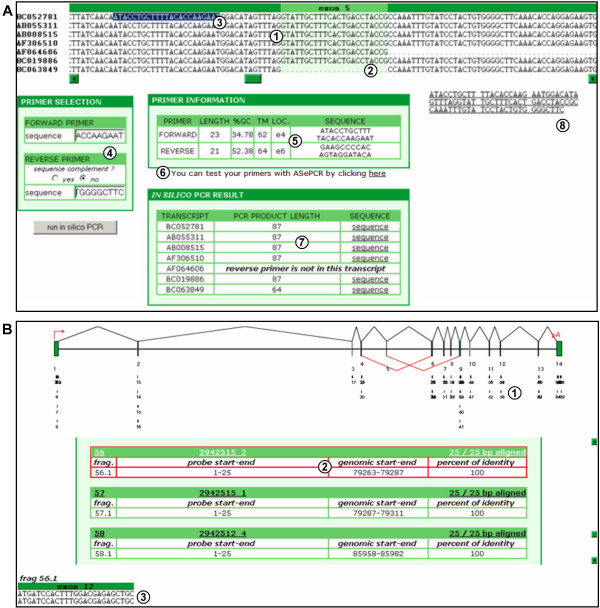
**"In silico PCR" and "Probe alignment" tools infast DB**. (A) The "*in silico *PCR" option in fast DB. (1) Multi-alignment of transcript sequences of the human *RANPB9 *gene, exon by exon. (2) Explicit skipping of exon 5 by the [GenBank:BC063849] transcript shown on multi-alignment. (3) Direct selection of primers on multi-alignment. (4) Form for inputting primer sequences. (5) General information regarding input primers: length, GC percentage, TM, location and sequence. (6) Link to test the user's primers with ASePCR. (7) Expected PCR product for each transcript with its length and the link to its sequence. (8) Sequence of one expected PCR product, clickable in order to display the restriction enzyme cut sites for this sequence. (B) The "probe alignment" option in fast DB. (1) Graphical representation of probe sequences aligned with the genomic exon/intron structure. (2) Table with alignment information for all aligned probe sequences; data regarding the selected probe are highlighted in red. (3) Alignment of the selected probe sequence with the genomic sequence.

Fast DB provides several tools to facilitate experimental studies of transcript expression. The "*in silico *PCR" tool (Figure 7A) displays a multi-alignment of all the different transcripts of a given gene that represents their common and specific sequences. Therefore, it becomes easy to design probes for downstream experimental applications, particularly PCR amplification. Thanks to the fast DB *in silico *PCR, the user can in a few minutes design primers for PCR amplification of all the transcripts as a single PCR product, or a specific SV, or all SVs or subsets thereof that give rise to PCR products of different sizes. For example, Figure 7A explicitly shows the skipping of exon 5 in the [GenBank:BC063849] transcript on the multi-alignment (Figure 7A, item 2). The primer sequences can be selected directly from this multi-alignment (Figure 7A, item 3), then copied and pasted on the corresponding boxes (Figure 7A, item 4). Once the *in silico *PCR is run, general information regarding input primers is displayed, including length, GC percent, TM, location and sequence (Figure 7A, item 5). Fast DB provides the expected PCR product length from each SV (Figure 7A, item 6). After clicking on the corresponding link, the sequence of the PCR product is displayed (Figure 7A, item 7) and is clickable in order to display restriction enzyme cut sites for this sequence (provided by RestrictionMapper [45]). Fast DB also provides a link to ASePCR [46] that displays the predicted PCR products expected in various tissues.

Finally, to assist the interpretation of microarray results, the "probe alignment" tool graphically provides the location of any sequence within the gene exon/intron structure, such as probes used in microarrays. Input sequences can be located within an exon, an intron, an exon/intron or an intron/exon junction, or an exon/exon junction. In all cases, the input sequences must be at least 20 nucleotides long, but in the case of an exon/exon junction they must cover at least 16 nucleotides of each exon. The example displayed in Figure 7B corresponds to the alignment of all Affymetrix exon-array core-level probes for the human *RANBP9 *gene (69 sequences, each 25 nucleotides long). Each of these probes is represented on the chart (Figure 7B, item 1), and their genomic positions, percentage identities with the genomic sequence and alignment lengths are displayed in a table under the diagram. After clicking on a given probe on the scheme, corresponding data in the table are highlighted in red (Figure 7B, item 2), and the corresponding probe alignment with the genomic sequence is provided at the bottom of the page (Figure 7B, item 3).

### Forum and documentation

To provide interactive help in using fast DB, we set up a forum in the "Forum/Documentation" section where users are invited to post their comments. Furthermore, we developed a quick help section by assembling short explanations and legends of fast DB charts. A complete fast DB documentation is available in PDF and HTML formats in the "Forum/Documentation" section.

## Discussion

In addition to describing the transcripts produced by human and mouse genes, as already reported [4], the new release of fast DB now provides tools that analyze the putative function of these transcripts and the regulation of their expression and therefore achieves an advance in AS databases by allowing SVs for human and mouse genomes to be interpreted functionally. To do that, fast DB integrates information and tools for predicting the functional consequences of AS. Several other databases have assembled protein information or information regarding AS. To try to correlate AS events with their functional consequences, some AS databases integrate protein sequences to annotate splicing events [47,48], while others indicate translational product start positions, end positions and amino acid sequences corresponding to some SVs [5,31,49-51]. The SpliceNest database even shows all 6-frame predicted ORFs [52]. However, none of these databases integrates tools for predicting the functional consequences of a given AS event. To the best of our knowledge, fast DB is the first freely-available system to offer direct links for making interactive predictions of functional protein domains from alternative exons. The next objective in the development of fast DB will be to integrate structural protein domain analysis, as this kind of domain has been shown to be altered by AS [2,53].

Furthermore, the translation of a given transcript can be inhibited or degraded through the NMD pathway and/or miRNA targeting. Therefore, transcripts that are predicted to be targeted by the NMD pathway and/or miRNA are indicated in fast DB. Only two databases containing NMD data have previously been available. The first, called "NMD database", comprises yeast data based on Affymetrix chip analyses with several UPF gene deletion conditions. The second is the ASTRA database [31], which indicates transcripts potentially targeted by NMD. However, ASTRA only contains 5,751 human genes and NMD prediction is constructed only from full-length cDNA sequences from UniGene. Several other databases contain information on miRNA targets, but fast DB is the only AS database that integrates the miRNA target feature in its transcript catalogue.

To help predict the tissue specificity of AS, fast DB provides a tissue distribution chart for each splicing event. Several studies have identified tissue-specific AS events [15,54], and some AS databases present EST tissue-expression histograms [49,50]. ASAP even provides lists of tissue-specific AS events by comparing the number of ESTs in different tissues [51]. However, fast DB is the only tool that provides a tissue distribution histogram for each splicing and transcriptional event. A limitation of this bioinformatic approach based on EST is that the number of ESTs varies considerably among tissues.

In addition to providing tools for predicting the translation product, function and expression pattern of each SV, fast DB offers tools to assist in their experimental analysis. The "*in silico *PCR" option enables the user to design primers for PCR amplification easily. Furthermore, the "probe alignment" tool provides a clear visualization of the genomic alignment of any input sequences, such as probes used in microarrays. In particular, the fast DB probe alignment tool allows each Affymetrix "probe selecting region" to be graphically associated with a given region (i.e. either constitutive exon or alternative region).

## Conclusion

Much effort has been devoted to setting up the most complete transcript catalogue to date. This work is an ongoing project and researchers must sustain their effort by providing new SVs to fill public databanks. In order to apprehend the role and impact of this transcriptome diversity in gene function, biologists need tools that provide information about the potential function of each SV, as well as its expression pattern. In addition, the scientific community using genomic/transcriptomic databases is increasing in size and diversity, not least because splicing deregulation is involved in many diseases [55], and the use of gene expression studies is continually growing in clinical analyses. Therefore, bioinformatic web interfaces have to be as user-friendly as possible so that they are readily searchable and intelligible at a glance by the whole biomedical community. We think that fast DB has reached the goal of providing a large number of bioinformatic tools that facilitate the study of the regulation of human gene product expression and of integrating these tools in a user-friendly, attractive and interactive web interface.

## Availability and requirements

Fast DB is freely available for online use at .

To display all graphical representations of fast DB (except tissue distribution histograms), the user's web navigator has to contain a flash plugin (freely downloadable at ).

## Authors' contributions

PG built the database pipeline, designed the relational database scheme, developed the web interface, is responsible for its maintenance, and contributed to writing the manuscript. MD provided comments and suggestions about the features of the database and revised the subsequent drafts of this manuscript. MC integrated the miRNA/transcript interaction sites in the database and in the web interface. DA conceived the idea of the database, provided direction for its development and contributed to writing the manuscript.
